# Leucosceptoside A from Devil’s Claw Modulates Psoriasis-like Inflammation via Suppression of the PI3K/AKT Signaling Pathway in Keratinocytes

**DOI:** 10.3390/molecules26227014

**Published:** 2021-11-20

**Authors:** Ivanka K. Koycheva, Liliya V. Mihaylova, Monika N. Todorova, Zhivka P. Balcheva-Sivenova, Kalina Alipieva, Claudio Ferrante, Giustino Orlando, Milen I. Georgiev

**Affiliations:** 1 Department Plant Cell Biotechnology, Center of Plant Systems Biology and Biotechnology, 4000 Plovdiv, Bulgaria; vkoy4eva@abv.bg (I.K.K.); vasileva.l.vl@gmail.com (L.V.M.); mntodorova@yahoo.com (M.N.T.); sivenova_jivka@abv.bg (Z.P.B.-S.); 2 Laboratory of Metabolomics, Department of Biotechnology, Institute of Microbiology, Bulgarian Academy of Sciences, 4000 Plovdiv, Bulgaria; 3 Institute of Organic Chemistry with Centre of Phytochemistry, Bulgarian Academy of Sciences, 1113 Sofia, Bulgaria; kalina.alipieva@orgchm.bas.bg; 4 Department of Pharmacy, G. d’Annunzio University, 66100 Chieti, Italy; cferrante@unich.it (C.F.); giustino.orlando@unich.it (G.O.)

**Keywords:** psoriasis, keratinocytes, inflammation, verbascoside, leucosceptoside A, devil’s claw, in silico docking

## Abstract

Psoriasis is a chronic inflammatory skin condition characterized by abnormal keratinocyte proliferation and differentiation that is accompanied with dysregulated immune response and abnormal vascularization. Devil’s claw (*Harpagophytum procumbens* (Burch.) DC. ex Meisn.) tubers extract has been used both systemically and topically for treatment of chronic inflammatory diseases such as arthritis, osteoporosis, inflammatory bowel disease, among others. However, its potential mechanisms of action against psoriasis remains poorly investigated. The human keratinocyte HaCaT cell line is a well-accepted in vitro model system for inflammatory skin disorders such as psoriasis. The present study involved an exploration of the effect of biotechnologically produced *H. procumbens* (HP) cell suspension extract and pure phenylethanoid glycosides verbascoside (VER) and leucosceptoside A (LEU) in interferon (IFN)-γ/interleukin (IL)-17A/IL-22-stimulated HaCaT cells as a model of psoriasis-like inflammation. Changes in key inflammatory signaling pathways related to psoriasis development were detected by reverse transcription polymerase chain reaction and western blotting. Treatment with LEU, but not VER and HP extract improved psoriasis-related inflammation via suppression of the PI3K/AKT signaling in IFN-γ/IL-17A/IL-22-stimulated HaCaT cells. Our results suggest that LEU may exhibit therapeutic potential against psoriasis by regulating keratinocyte differentiation through inhibition of the PI3K/AKT pathway.

## 1. Introduction

Psoriasis is a chronic, inflammatory, immune-mediated skin pathology, affecting approximately 2–3% of the adult world’s population [1]. It is manifested mainly with psoriatic lesions and is hence associated with serious complications such as psoriatic arthritis, obesity, sleep apnea, depression and certain autoimmune diseases [1,2,3,4]. Chronic psoriasis is characterized with a deterioration in the quality of life. The disease has been recognized as a major global health burden by the World Health Organization, since 2014 [1,4].

The pathophysiology of psoriasis is characterized by hyperproliferation, dysfunctional apoptosis and abnormal differentiation of keratinocytes that are accompanied with infiltration of immune cells in the dermis and epidermis [1,5,6]. The percentage of proliferative cell populations are found to be increased in the basal and suprabasal layers in psoriasis. The marker of proliferation Ki67 together with excessive abundance of keratin 10 (K10) are reported to be overexpressed in psoriatic lesions [7,8]. Keratinocytes play a key role in initiating, maintaining and enhancing of the immune and inflammatory responses associated with psoriasis. The complex network formed from the interaction of keratinocytes, dendritic cells, vascular endothelial cells and T helper (Th) cells (predominantly Th1 and Th17) contribute to development of psoriasis [2,9,10]. Subsequently, the secretion of inflammatory cytokines such as tumor necrosis factor alpha (TNF-α), interferon gamma (IFN-γ), interleukin- (IL) 6, IL-17A and IL-22 elicits psoriasis inflammation that results in keratinocytes upregulation of neutrophil, attracting chemokines such as [(C-X-C motif) ligand-) CXCL1, CXCL2, CXCL3, CXCL5, CXCL8, (C-C motif) ligand-) CCL2, CCL20], antimicrobial peptides (lipocalin-2, β-defensins, S100 calcium-binding proteins) and immunomodulatory molecules such as intercellular adhesion molecule. In addition, the activated psoriatic keratinocytes respond with deranged proliferation and differentiation programs [6,11,12,13,14]. Signaling pathways, known to be implicated in psoriasis development, are the Janus kinase/signal transducer and activator of transcription (JAK/STAT), the nuclear factor kappa B (NF-κB) and the phosphatidylinositol 3-kinase/protein kinase B/mammalian target of rapamycin (PI3K/AKT/mTOR) pathway [1,5,15,16,17,18].

The approved anti-psoriatic therapy lines include drugs from the following classes: (i) topical agents such as corticosteroids, retinoids, vitamin D analogues and calcineurin inhibitors; (ii) systemic drugs such as corticosteroids, biological drugs, including monoclonal antibodies against TNF-α (etanercept, infliximab, adalimumab), IL-17A (secukinumab, ixekizumab), IL-17RA (brodalumab) and IL-12/-23 (ustekinumab, guselkumab, risankizumab); (iii) photo-based therapies; or iv) combinations between medications of different classes [1,3,19]. Severe adverse drug reactions (i.e., hypertension, nephrotoxicity, hepatotoxicity and hyperlipidemia) limit the use of current therapy and expose the need of new treatment strategies and development of safer and more effective anti-psoriatic agents. Several advantages, such as promising anti-psoriatic efficiency, relatively low toxicity and the possibility to be included in novel drug delivery systems position natural products and plant-derived drug leads as a favorable option for psoriasis management [20]. The ethnopharmacological approach can open up a new perspective for natural products utilization as an adjuvant to the approved therapy, especially concerning the topically applied anti-psoriatic agents. Such hybrid combinations could further synergistically alleviate skin inflammation, reduce the dose of synthetic medicine(s) and decrease the observed adverse reactions. Experimental data from recent years confirm the potential in psoriasis management of medicinal plants such as *Aloe vera* L. Burm.f., *Centella asiatica* (L.) Urb., *Curcuma longa* L., *Lavandula angustifolia* Mill., *Mahonia aquifolium* (Pursh) Nutt., *Psorospermum febrifugum* Spach, *Strobilanthes cusia* (Nees) Kuntze [21,22,23].

*Harpagophytum procumbens* (Burch.) DC. ex Meisn. (hereafter called HP; common name Devil’s claw) is an herbaceous perennial plant, which is native to the Kalahari Desert. The tubers of the plant exhibit strong anti-inflammatory properties, attributed mainly to the content of iridoid glycosides, i.e., harpagide and harpagoside, and hence are considered as an alternative to the non-steroidal anti-inflammatory drugs. The plant has been traditionally used for thousands of years to treat wide range of ailments, including pain and inflammation [24]. Nowadays, due to its significant pharmacological potential HP extract is implemented in the treatment of inflammation and oxidative stress-related diseases, including arthritis, osteoporosis, inflammatory bowel disease, low-back pain, diabetes, neurodegenerative diseases, tendonitis, kidney inflammation and heart diseases [24,25,26,27,28,29,30,31]. Apart from the main bioactive iridoids that are sufficiently investigated for their mechanism of action, the HP extracts are an abundant source of phenylethanoid glycosides that are of great interest as potential anti-inflammatory compounds. For instance, verbascoside (VER, also known as acteoside), is the main phenylethanoid glycoside found in HP extracts and demonstrates anti-inflammatory, antiviral, antibacterial, antioxidant, neuroprotective and hepatoprotective properties [24,26,32,33,34]. Leucosceptoside A (LEU) is structurally similar, although less explored than verbascoside, and is reported to possess promising antioxidant and anti-inflammatory activities [24,26,32,34]. Here, we have employed specific HP cell suspension line that is selected in our lab and is characterized by its abundance of phenylethanoid glycosides. Further, the HP cell suspension extract has been found to lack harpagide and harpagoside in contrast to the extracts from mother plant [35].

The present study aimed at NMR-based profiling of a biotechnologically produced HP cell suspension extract, rich in phenylethanoid glycosides. Molecular docking calculations predicted the affinity between the selected Devil’s claw metabolites and protein structures, involved into psoriasis pathophysiology. The biological activity of HP extract, VER or LEU was further evaluated in an in vitro model system of a psoriasis-like inflammation in HaCaT keratinocytes. Modulation in the relative mRNA expression and protein levels of key mediators of psoriasis-related inflammation was examined in order to clarify the signaling pathways affected by the tested extract and its metabolites in human keratinocytes by combining several approaches. Potential molecular mechanism of the anti-psoriatic action of HP extract, VER and LEU was proposed.

## 2. Results

### 2.1. Phytochemical Analysis of H. procumbens Cell Suspension Extract

**Figure 1:**
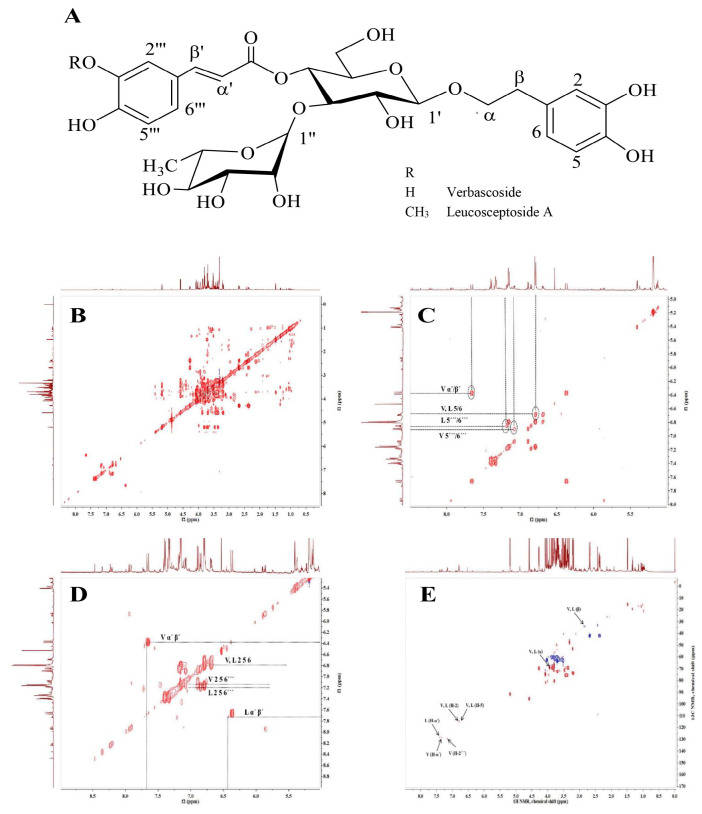
Chemical structure of verbascoside and leucosceptoside A (**A**); Total correlation spectroscopy (^1^H-^1^H TOCSY) and NMR spectra of *H. procumbens* (HP) cell suspension extract (**B**), the aromatic part of the spectra (**C**); ^1^H-^1^H homonuclear correlation spectroscopy (COSY) NMR spectra of HP cell suspension extract (**D**) and ^1^H-^13^C HSQC NMR signals (**E**).

The ^1^H and 2D (J-resolved, COSY, HSQC and TOCSY) NMR data were used to elucidate structures of the main secondary metabolites VER and LEU in HP cell suspension extract. The ^1^H NMR and HSQC signals of VER revealed the presence of two sets of ABX systems: H-2 (δH 6.80, d/δC 118.52), H-5 (δH 6.79, d/δC 118.11) and H-6 (δH 6.69, dd/δC 124.19) assigned to 3,4-dixydroxy-β-phenyletoxyl moiety and H-2′ (δH 7.16, d/δC 117.17), H-5′ (δH 6.89, d/δC 118.65) and H-6′ (δH 7.08, dd/δC 121.90) corresponded to the second ABX system of the caffeoyl moiety. The signals of two trans-olefinic protons α′ (δH 6.37, d/δC 112.80) and β′ (δH 7.66, d/δC 146.7) each J = 16.0 Hz, indicated the double bond with the E-configuration of the caffeic acid unit. In addition, the disaccharide structure has been indicated by the two anomeric signals of H-1′ (δH 4.49, d/δC 105.57) and H-1′ (δH 5.13, s/δC 104.41) attributed to the β-glucose and α-rhamnose units [36,37]. The chemical shifts of LEU were similar to that of VER, except the additional signal of methoxy group at δH 3.90 (3H, s/δC 60.19) suggested that the acyl moiety is ferulic acid [38]. The structures of both compounds were confirmed by the correlations in the ^1^H-^1^H COSY and TOCSY spectra (Figure 1).

In the current experimental set-up, 10.04 g HP dry cell biomass was used to produce 3.68 g of dry extract. The content of VER and LEU in the HP cell biomass and the extract is available in Supplementary Table S1.

### 2.2. In Silico Docking Simulation

**Figure 2:**
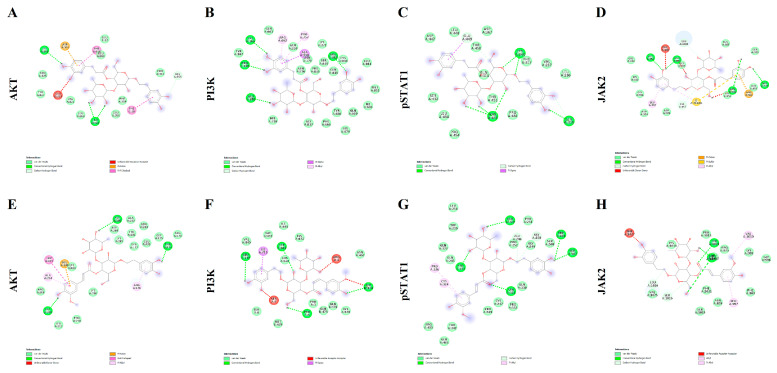
Putative molecular interactions between verbascoside (VER) and AKT (**A**); VER and PI3K (**B**); VER and pSTAT1 (**C**); VER and JAK2 (**D**); leucosceptoside A (LEU) and AKT (**E**); LEU and PI3K (**F**); LEU and pSTAT1 (**G**); LEU and JAK2 (**H**).

Molecular docking approach was utilized with the aim to predict the putative affinities between the phytochemicals VER and LEU with the following proteins: AKT; PI3K; pSTAT1 and JAK2. The interactions of the selected pure compounds with the aforementioned target proteins were expressed in terms of binding free energy (_∆_G) and binding affinity constant (Ki; Table 1), and pointed to putative sub-micromolar affinity (0.1–5.4 µM). In this regard, different binding interactions, including Van der Waals, hydrogen and pi bonds were predicted (Figure 2); thus, further suggesting direct interactions between the studied pure compounds (VER and LEU) and the target protein structures. Details of the aforementioned interactions are supplied in the supplementary file (Supplementary Figures S2–S9).

### 2.3. H. procumbens Extract and Its Constituents VER and LEU Modulate the Gene Expression Profile of IFN-γ/IL-17A/IL-22-Stimulated Keratinocytes

The transcription factor NF-κB plays a major role in skin inflammation, as well as, in cellular survival, proliferation and differentiation and hence its activation appears detrimental for the pathogenesis of psoriasis [39]. Concomitantly, the JAK/STAT signal transduction pathway and the PI3K/AKT/mTOR signaling pathway are also involved in psoriasis development and progression [13,40,41,42,43]. The PI3K/AKT/mTOR activation and STAT3 signaling are known to promote acanthosis, which is implicated in the immunopathogenesis of psoriasis [5,16]. In this context, we investigated the effect of HP extract, VER and LEU on the expression of genes related to above mentioned molecular pathways, by the means of the RT-qPCR analysis.

**Figure 3:**
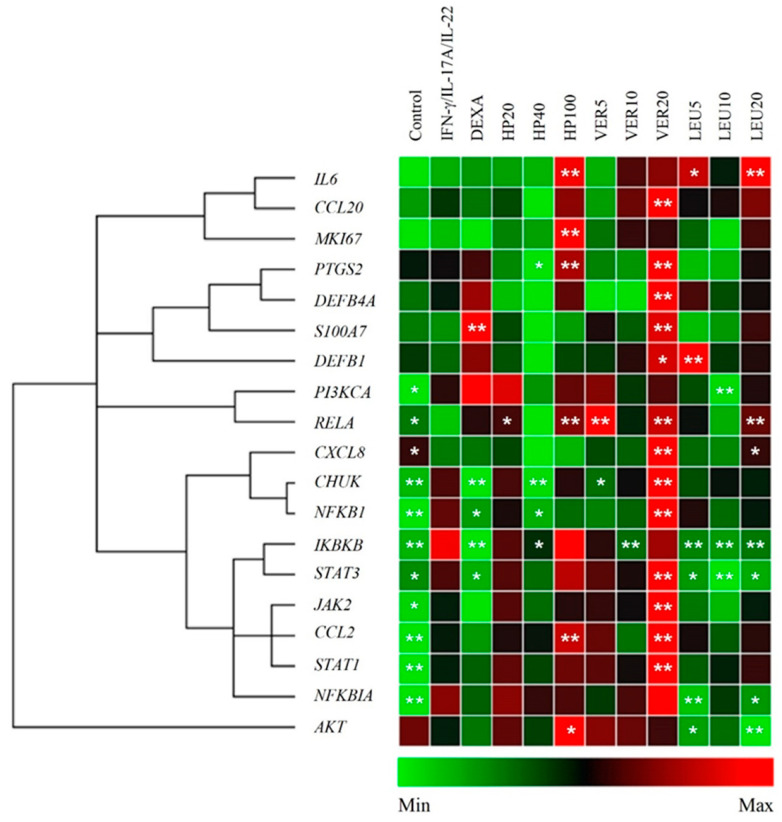
*H. procumbens* (HP) extract and pure leucosceptoside A (LEU), but not verbascoside (VER), balance inflammatory gene expression in the in vitro psoriasis-like model. The clustergram and heatmap of the relative gene expression analysis from the RT-qPCR. Data are representative from three independent experiments (mean ± SEM). * *p* < 0.05 and ** *p* < 0.01 compared to the psoriasis-like model group.

The data obtained upon HP extract, VER and LEU treatment are presented on Figure 3. Significant upregulation of the genes related to NF-κB, JAK/STAT and PI3K/AKT signaling pathways, as well as, the inflammatory factor CCL2 (encoding the MCP1 protein) were observed as a result of the IFN-γ/IL-17A/IL-22 stimulation of HaCaT keratinocytes. Overexpression of *CHUK*, *IKBKB*, *NFKBIA*, *NFKB1*, *JAK2*, *STAT1* and *STAT3*, as inflammation related factors, was detected in the psoriasis model group. These elevated expression levels appeared consistent with previous studies that employed a similar IFN-γ/IL-17A/IL-22-induced psoriasis-like model in keratinocytes [6,11,13,23]. The IL-17A upregulates keratin 17 (strongly expressed in psoriatic lesions) through STAT1- and STAT3-dependent mechanisms [44]. The STAT1, STAT3 and NF-κB are known to play a key role of the transcriptome network implicated in the psoriasis development [17,45]. Positive transcriptional regulation of expression of approximately 400 genes by predominant IFN-γ signature, dependent on STAT1, is detected in psoriatic skin lesions [6]. Similarly, to our previously reported data [23], *STAT1* appeared more affected in the model than *STAT3* (the difference in expression was over 5 folds) upon stimulation with IFN-γ/IL-17A/IL-22.

Treatment with HP extract at concentration of 40 µg/mL inhibited the genes from NF-κB signaling (*CHUK*, *IKBKB* and *NFKB1*) at mRNA level. This appeared accompanied by an increase in the relative mRNA expression of *RELA* and *NFKBIA*. Collectively, these results indicate activation of NF-κB signaling pathway and hence correspond to positive regulation of *CCL2*, *IL6* and *PTGS2* (encoding COX-2) expression. Interestingly, HP extract acted bi-directionally on the mRNA expression of *PTGS2*, as at concentration of 40 µg/mL downregulated the *PTGS2* expression, while at the highest (100 µg/mL) significantly increased it. At the same time HP extract at its highest concentration upregulated *MKI67* and *AKT* mRNA expression. This upregulation was not observed upon LEU application.

Intriguingly, the tested pure compounds VER and LEU, having similar chemical structures (Figure 1A), exhibited different biological activity in psoriatic keratinocytes. Sharp upregulation of the mRNA levels of nearly all studied genes [*CCL2*, *CCL20*, *IL8*, *PTGS2*, *S100A7*, beta defensin (*DEFB*)*1*, *DEFB4A*, *CHUK*, *RELA*, *NFKB1*, *JAK2*, *STAT1* and *STAT3*] upon VER treatment was detected at the highest concentration used (20 µM). Despite the fact that VER possesses anti-inflammatory properties and inhibits NF-κB signaling in various tissues [24,32] in the present model system, it produced rather pro-inflammatory effect in psoriatic keratinocytes. The other phenylethanoid glycoside LEU suppressed the mRNA expression of *IKBKB* and *NFKBIA* and increased *RELA* mRNA expression at its highest concentration employed (20 µM). Therefore, we could speculate that LEU modulates the NF-κB signaling pathway.

The chronic epidermal hyperplasia in psoriatic lesions is due to keratinocytes proliferation and main events of the immune response expressed by overactivation of PI3K/AKT/mTORC1 and STAT3 signaling cascades [46,47]. The apoptosis induction was reported as a key mechanism for regression of psoriatic hyperplasia with conventional UVA and methotrexate therapy [47,48]. The PI3K/AKT/mTOR cascade plays a role on the resistance of psoriatic keratinocytes to apoptosis and dysregulation in keratinocyte differentiation [5,46,49,50]. Here, the IFN-γ/IL-17A/IL-22 stimulation in HaCaT cells increased the PI3K/AKT related genes and upregulated Ki67, which is amongst the important markers of hyperproliferation in psoriatic skin. Treatment with LEU resulted in decreased *PI3KCA* mRNA expression, while the expression of *AKT* was downregulated by both VER and LEU. However, both phenylethanoid glycosides did not influence the expression of proliferation marker Ki67. In contrast, HP extract enhanced *MKI67* mRNA expression, which is supposed to boost the cell proliferation. Moreover, we found that the *STAT3* gene expression appeared inhibited by LEU treatment at all tested concentrations. STAT3 together with NF-κB regulates the expression of genes that control cell survival and proliferation, and inhibition of these transcription factors is a promising therapeutic strategy in psoriasis [39].

Together, these results demonstrated that HP extract affected mainly NF-κB-related genes and their activation points towards induction in the immune response and thereby obstructs keratinocytes hyperproliferation. Pure VER appeared to influence on the genes from both NF-κB and JAK/STAT signaling pathways. In addition, HP extract activated *AKT* mRNA expression while LEU suppressed *STAT3*, *PI3KCA* and *AKT* in stimulated keratinocytes. In summary, our findings demonstrate that LEU targets keratinocytes by suppression of PI3K/AKT signaling. Moreover, the inhibition in the overactivated *AKT* gene expression suggests that LEU could modulate the keratinocyte differentiation program in psoriasis.

### 2.4. Effect of H. procumbens Extract and Pure VER and LEU on Expression of Key Psoriasis-Related Proteins

**Figure 4:**
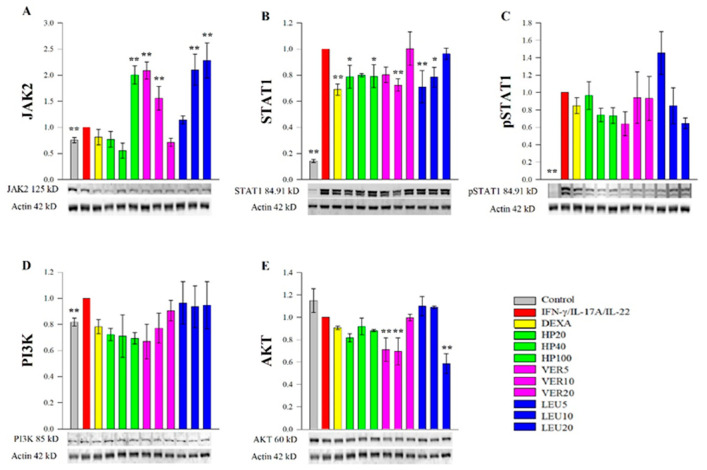
*H. procumbens* extract (HP), pure verbascoside (VER) and leucosceptoside A (LEU) effect on the key psoriasis-related proteins. Protein abundance of JAK2 (**A**), STAT1 (**B**), phosphorylated STAT1 (**C**), PI3K (**D**) and AKT (**E**) obtained from the Western blotting analysis normalized over β-actin. Data (mean ± SEM) are representative from three independent experiments. * *p* < 0.05 and ** *p* < 0.01 compared to the psoriasis-like model group.

To complement the data from the RT-qPCR analysis and to elucidate the underlying molecular mechanisms of action of HP, VER and LEU we further examined the protein abundance of key transcription factors, involved in psoriasis pathogenesis. Our experimental results revealed that JAK2, STAT1 and PI3K were activated, while AKT was not influenced at a protein level in the psoriasis-like model. The results from the Western blot analysis (Figure 4) demonstrated suppressive effects of HP extract, VER and LEU on the total STAT1 protein (Figure 4B), while the level of pSTAT1 was moderately affected upon the LEU treatment (Figure 4C). In comparison to the data obtained from the RT-qPCR analysis (Figure 3), the *STAT1* gene expression level was not influenced upon either the HP or the phenylethanoid glycosides treatments. Further, JAK2 was evaluated at protein level (Figure 4A) and HP extract (100 µg/mL), LEU (10 and 20 µM) and VER (5 and 10 µM) increased its abundance. In parallel with RT-qPCR data for *JAK2* (Figure 3), VER at its highest concentrations positively regulated *JAK2* mRNA expression about 5 folds, while LEU treatment did not influence *JAK2*.

The protein level of PI3K was not affected upon HP extract, VER or LEU treatment in stimulated keratinocytes (Figure 4D) while AKT abundance appeared inhibited from VER (5 and 10 µM) and LEU (20 µM) in the stimulated HaCaT cells (Figure 4E). These data confirmed the suppressive effect (especially of LEU) on AKT signaling represented by downregulation of its gene expression, suggesting possible independent of PI3K interaction.

Collectively, these findings indicate that the biotechnologically produced HP cell suspension extract and the phenylethanoid constituents (VER and LEU) affect JAK2/STAT1 signaling pathway in psoriatic keratinocytes. Moreover, both VER and LEU could eventually interfere with the PI3K/AKT signaling pathway at a protein level.

### 2.5. Proposed Mechanism of the Anti-Psoriatic Action of H. procumbens Extract, VER and LEU in Keratinocytes

**Figure 5:**
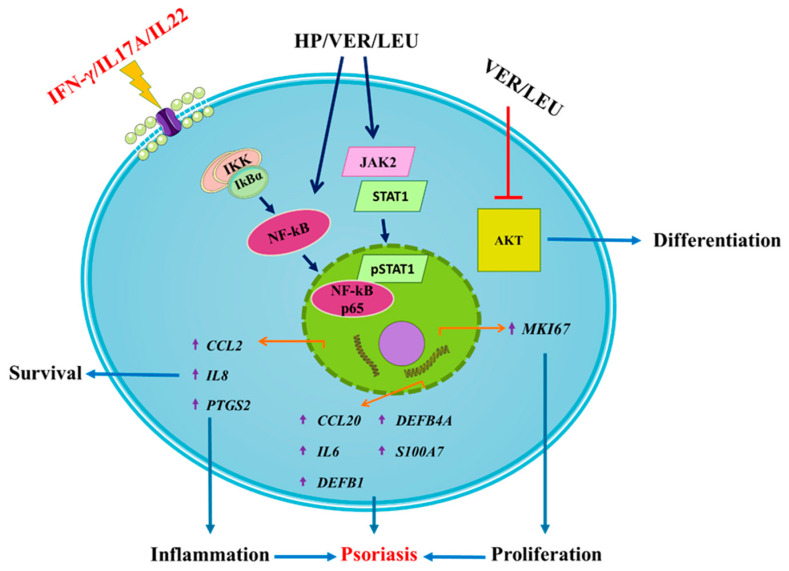
Proposed mechanism of action of *H. procumbens* extract (HP), pure verbascoside (VER) and leucosceptoside A (LEU) on the transcriptional regulation of genes related to inflammation and psoriasis in IFN-γ/IL-17A/IL-22-stimulated keratinocytes. The HaCaT cells exposed to the combination of pro-inflammatory cytokines respond with activation of NF-κB, JAK2/STAT1 and PI3K/AKT signaling pathways. Activation of JAK2 upon cytokine stimulation leads to STAT1 activation and its subsequent phosphorylation. Following NF-κB activation and nuclear translocation the phosphorylated STAT1 transcriptional activation of psoriasis-related inflammatory genes in the activated keratinocytes is induced (e.g., *IL6*, *IL8*, *CCL20*, *CCL2*, *PTGS2*, *DEFB1*, *DEFB4A* and *S100A7*). Simultaneously, the activation of the PI3K/AKT axis in psoriatic keratinocytes correlates with induction of hyperproliferation, disrupted differentiation and aggravation of the inflammatory milieu. The phenylethanoid glycosides VER and LEU both interfere with the psoriasis-related inflammation through suppression of the AKT signaling.

Data of HP extract, VER and LEU treatment in IFN-/IL-17A/IL-22-induced psoriasis-like inflammation targeting various signaling events involved in the psoriasis pathogenesis are summarized on Figure 5. Surprisingly, results showed HP extract and VER have demonstrated stimulatory rather than anti-inflammatory effect in human keratinocytes by activating the NF-κB and JAK2/STAT1 signaling. Further, we also observed that both VER and LEU could inhibit AKT signaling, suggesting potential modulation of keratinocyte proliferation and differentiation in psoriasis.

## 3. Discussion

In the present study, we have provided evidence for psoriasis-like changes in human keratinocytes as a result of IFN-γ/IL-17A/IL-22 stimulation. Moreover, we investigated the activity of biotechnology produced HP extract and its phenylethanoid constituents (VER and LEU) in the in vitro psoriasis-like model. The underlying molecular mechanisms of action of the cell suspension extract and of its pure compounds were eventually elucidated.

Inflammation plays an essential role in the development of psoriasis and hence keratinocytes’ inflammation appears distinctive mark [51]. The transcription factor NF-κB has been reported to be implicated in the psoriasis induction [10,40,52]. The inflammatory response, NF-κB activation and production of pro-inflammatory mediators (IL-1β, IL-6, TNF-α, IFN-γ, CXCL8, CCL2) are strongly associated with disease pathogenesis [40]. The complex NF-κB signaling consists of p50, p52, RelA (p65), RelB and c-Rel subunits that are sequestered in the cytoplasm in an inactive form and binding to members of inhibitor of κB (IκB). Inflammatory stimulation phosphorylates and degrades IκB by IκB kinase (IKK) complex that leads to release and nuclear translocation of the p65 subunit, which in turn activates the canonical NF-κB pathway. Activation of p65 triggers the secretion of IL-8 and CCL20 and complicates the inflammatory response [51]. Treatment with HP extract and VER increased expression of *IL8*, *PTGS2* and *CCL2*, *CCL20* via NF-κB activation. This suggests boosting of immune response and that HP extract and VER application could accelerate psoriasis-like inflammatory environment. In contrast, LEU was not involved in such regulation.

The inhibition of NF-κB and reduction of downstream regulated cytokines has been proven as a mechanism of action for the anti-inflammatory and anti-psoriatic effects of a number of extracts, fractions or pure compounds in vitro and in vivo [42,53,54]. Blocking the NF-κB signaling and TNF-α (its canonical pathway inducer) is one of successful approaches of the anti-psoriasis treatment [40]. In this context, it is seen from the results that HP extract and pure VER and LEU demonstrated stimulatory effects in our model, promoting production of pro-inflammatory mediators and proliferation of keratinocytes via NF-κB activation. These findings support the hypothesis that HP extract’s activity is mainly attributed to the VER and LEU content. An advantage of the extract over pure molecules could be that due to the content of a variety of chemical components in it affects the cell response through different mechanisms. Additionally, VER application resulted in enhanced mRNA level of markers of psoriasis (*S100A7*, *DEFB1*, *DEFB4A*).

Similarly, Zhang et al. [55] reported that CD100-Pplexin-B2 complex promotes the inflammation in psoriasis by activating NF-κB in IFN-γ/IL17A/IL22-stimulated primary keratinocytes. Transglutaminase 2 mediates the upregulation of cytokines and chemokines (IL6, CXCL8 and CCL20) via activation of NF-κB in imiquimod treated keratinocytes [56]. Chemoattractant CCL20 recruits dendritic and T cells stimulating inflammation in the skin and was upregulated in psoriasis and was produced under control of NF-kB signaling in keratinocytes [40]. Steroids have essential role in the treatment of autoimmune and inflammatory diseases. Potent anti-inflammatory effect of glucocorticoid therapy was achieved through NF-κB inhibition. Downregulated expression of genes related to NF-κB and *CCL2*, *CCL20* was expected upon DEXA treatment in activated keratinocytes [57], which was also detected in our study. Directly activated expression of CCL20 through DEXA treatment in keratinocytes deteriorated skin conditions such as perioral dermatitis and rosacea [58].

In this case, VER application may amplify psoriasis-associated inflammation due to its mechanism of NF-κB activation and positive expression of *CCL20*, among others. Most of the IFN-γ-induced effects in keratinocytes are strengthened by TNF-α through activation of transcription factor NF-κB, which regulates gene expression frequently with STAT1 cooperation [6]. In the present study, CCL20 and NF-κB signaling were not influenced upon LEU treatment.

Other transcriptional pathway that takes part in inflammatory and immune-mediated disorders is JAK/STAT pathway, which is involved in rheumatoid arthritis, psoriasis and inflammatory bowel disease, and transduces downstream of cytokines critical to the pathogenesis of psoriasis [15,59]. Activation of JAK/STAT is characteristic and strongly expressed in psoriatic disease, hence JAK inhibitors (i.a., baricitinib, ruxolitinib, upadicitinib, cerdulatinib) have established therapeutic effects in psoriasis [15,59,60]. Anti-inflammatory activity through inhibition of NF-κB and JAK/STAT signaling was reported both in vitro and in vivo [13,17,41,42]. In addition, STAT3 phosphorylation was suppressed along with the positive regulation of JAK2 and JAK3 [52]. Correspondingly, in our experimental data JAK2 protein expression was upregulated while phosphorylation of STAT1 was not influenced upon HP extract, VER and LEU application in IFN-γ/IL-17A/IL-22-stimulated keratinocytes. These findings suggest that they demonstrate pro-inflammatory activity in our psoriasis-like model.

Studies have reported hyperactivity of AKT/mTOR and its downstream molecular target ribosomal protein S6 kinase 1 (S6K1) in the skin lesions of patients with psoriasis [49,50], and the highly active PI3K/AKT/mTOR pathway can lead to the abnormal differentiation of keratinocytes, which is generally recognized as a main pathological feature of psoriasis [16,46]. Inactivation of PI3K/AKT signaling provides a novel approach in psoriasis management [61]. The classical PI3K/AKT pathway leads to activation of intracellular signaling that regulates cell metabolism, growth, proliferation, differentiation and migration [18,43,62]. Among malignant skin disorders, dysregulation of PI3K/AKT pathway is also associated with acne and psoriasis. The Th1/Th2/Th17 imbalance activates PI3K/AKT/mTOR pathway and results in the occurrence and development of psoriasis. The PI3K binds to AKT and thereby in turn activates the mTOR, which promotes keratinocyte hyperproliferation and inhibits differentiation [18]. Inactivation of PI3K and AKT pathways reduced proliferative effects in psoriatic keratinocytes and improved psoriasis-like pathologies in vitro and in vivo [14,60,63]. Downregulation of characteristic for psoriasis Ki67 and psoriasin in reconstituted psoriatic skin organoid culture indicated alleviation of inflammation and hyperproliferation [7]. Similar study of Thatikonda et al. [43] observed a decrease in the proliferative marker Ki67 through inhibition of AKT signaling. We, hence, speculate that AKT signaling inhibition observed upon VER and LEU treatment could lead to managed proliferation of activated HaCaT cells. However, our results showed that the HP extract increased expression of Ki67, while its levels were kept as equal as the control group from VER and LEU treatment. Collectively, further experiments are needed to identify the exact targeting site of AKT.

## 4. Materials and Methods

### 4.1. Materials

VER (molecular weight 624.59 g/M; purity ≥ 95%; #4994 S) was supplied from Extrasynthese (Genay, France). LEU (molecular weight 638.61 g/M) was isolated by applying the HP crude extract to Merck Lobar columns (RP-8 and RP-18), further eluted with a water/methanol gradient and identified by ^1^H NMR (600 MHz) and ^13^C NMR (150 MHz) as described early by Georgiev et al. [24]. Deuterated methanol and water were delivered from Deutero GmbH (Kastellaun, Germany). Cell culture media (#D5796), fetal bovine serum (#F7524), MTT reagent (#M21281), antibiotic-antimycotic solution (#A5955), trypsin-EDTA (#59418C), dexamethasone (DEXA; #D1756), Bradford reagent (#B6916), RIPA lysis buffer (#R0278), protease and phosphatase inhibitor cocktail (#PPC1010), RNAzol RT reagent (#R4533), standard chemicals and substances were of analytical grade and were obtained from Merck KGaA (Darmstadt, Germany). The recombinant human IL-17A (#ENZ-PRT188) and IL-22 (#ENZ-PRT250) were purchased from Enzo Life Sciences AG (Lausen, Switzerland) and IFN-γ (#10067-IF) was from R&D Systems (Minneapolis, MN, USA). Buffers and reagents for electrophoresis, immunoblotting and RT-qPCR were delivered from Bio-Rad Laboratories, Inc. (Hercules, CA, USA). Primary rabbit antibodies employed for Western blotting: anti-AKT (#9272), anti-JAK2 (#3230S), anti-PI3K (#4257), anti-STAT1 (#14994) and anti-phospho-STAT1 (#7649) were acquired from Cell Signaling Technology (Leiden, The Netherlands). Direct hFAB Rhodamine anti-actin beta antibody (#AHP2417) and secondary antibody used for fluorescent detection anti-rabbit IgG Star-Bright Blue 700 (#12004162) were both from Bio-Rad (Hercules, CA, USA).

### 4.2. Cultivation and Extraction of H. procumbens Cell Suspension

The HP suspension culture was cultivated as described earlier [35] on a liquid Linsmayer and Skoog nutrient medium, supplemented with 0.2 mg/L 2,4-dichlorophenoxyacetic acid and 30 g/L sucrose, at 26 °C, in the dark, on an orbital shaker, at 100 rpm. After cultivation, the cell biomass was frozen and freeze-dried, followed by extraction with 50% aqueous methanol under sonication, at room temperature (RT), for 20 min. Further, the obtained extract was filtrated and concentrated via rotary vacuum evaporator at 40 °C, freeze-dried and stored at −20 °C prior to chemical analysis and biological assays.

### 4.3. Nuclear Magnetic Resonance (NMR)-Based Metabolite Profiling and Chromatographic Analysis

The NMR analysis was conducted as previously described by Koycheva et al. [23]. Ten (10) mg of *H. procumbens* cell suspension extract was dissolved with equal amounts (0.4 mL each) of CD_3_OD-*d4* (99.8%) and D_2_O (99.9%), buffered with KH_2_PO_4_ (pH = 6), containing 0.01% TSPA-*d4* as an internal standard. The sample was homogenized on vortex for 1 min, sonicated for 20 min (35 kHz at RT), followed by centrifugation (12,000 rpm at 4 °C) for 20 min. The obtained clear supernatant was transferred into 5 mm NMR tube. The ^1^H NMR and 2D NMR spectra were recorded at 25 °C on an AVII+ 600 spectrometer (Bruker, Karlsruhe, Germany), operating at a frequency of 600.01 MHz with relaxation time of 4.07 s. The acquired spectra were manually phased and baseline corrected, and processed further by using MestReNova software (version 12.0.0, Mestrelab Research, Santiago de Compostela, Spain). The VER and LEU were identified by NMR data analysis, comparison with spectra of authentic samples and previously published data [36,37,38].

Both VER and LEU were quantitative analyzed by high-performance liquid chromatography (HPLC) and data were presented as mg/g dry extract. The HPLC system (Waters, Milford, MA, USA) consisted of a binary pump, dual wavelength (λ) absorbance detector and a reverse-phase column (Kinetex^®^ C18, 100 Å core-shell column, Phenomenex, Torrance, CA, USA), controlled by Breeze software version 3.30 SPA from Waters was used. The VER and LEU measurement was based on an HPLC protocol previously used by Georgiev et al. [64]. In brief, the mobile phases used for metabolites separation were methanol (phase A) and water (phase B) with the following gradient: change of phases A from 10 to 50 (0–10 min, flow rate of 1.0 mL/min), from 50 to 100 (10–15 min, flow rate of 1.1 mL/min), from 100 to 10 (15–20 min, flow rate of 0.8 mL/min), followed by a linear gradient to 0 (20–25 min, flow rate of 1.0 mL/min). All analyses were conducted at 26 °C and 330 nm detection wavelength.

### 4.4. Cell Culture and Treatment

The HaCaT keratinocytes were purchased from Cell Line Service GmbH (Eppelheim, Germany). Cell line was cultured following the previously described conditions [23]. The psoriasis-like inflammatory milieu was achieved by exposing near confluent HaCaT cells to the combination of IFN-γ/IL-17A/IL-22 (at a final concentration of 1 ng/mL each) [23,65] for 1 h and further treated either with HP extract (20, 40, 100 µg/mL), VER and LEU (both at 5, 10, 20 µM), DEXA (5 µM) or vehicle alone (0.2% DMSO). The concentration range of the HP extract and its active compounds were determined following evaluation of their effect on the cell viability of HaCaT cells. Exposure to different concentrations of HP extract, VER and LEU for 24 h did not affect remarkably the cell viability up to 100 μg/mL for the extract and 100 μM for the pure compounds, respectively (Supplementary Figure S1). A positive control, DEXA, was used. Samples for subsequent analyses were collected as follow: total RNA and protein lysates at the 6th and 24th hour from treatment, respectively. Each experiment was performed in triplicate (at least).

### 4.5. In Silico Molecular Docking

The crystal structures of the key proteins assayed in the present study were derived from Protein Data Bank (PDB, www.wwpdb.org accessed on 1 April 2021). The employed PDB IDs were: 6BBV for JAK2; 1BF5 for phosphorylated STAT1 (pSTAT1), 1O6L for AKT, 5ITD for PI3K. Regarding the docking calculations, the full protocol is described previously [66].

### 4.6. Quantitative Real-Time Polymerase Chain Reaction (RT-qPCR)

The total RNA isolation, synthesis of cDNA and the relative expression of target genes were performed as described by Koycheva et al. [23]. The primer sequences used are listed in Supplementary Table S2.

### 4.7. Western Blot Analysis

The RIPA buffer for cell protein extraction, Bradford assay and Western blot analysis (the buffers, membranes and primary rabbit antibodies against AKT, JAK2, PI3K, STAT1, pSTAT1) were described elsewhere [23]. Normalization was done over β-actin as a housekeeping protein. The ChemiDoc MP imaging system (Bio-Rad, Hercules, CA, USA) and Image Lab software 6.0.1 (Bio-Rad, Hercules, CA, USA) were utilized to visualize and quantify the immunodetection.

### 4.8. Statistical Analysis

The data were statistically processed with SigmaPlot software 11.0 (Systat Software GmbH, Erkrath, Germany) and obtained results are presented as mean ± SEM. The determination of the differences between groups was analyzed by Student’s t-test or one-way analysis of variances (ANOVA) with Bonferroni’s post hoc test. Value of *p* < 0.05 was defined as a threshold for significance.

## 5. Conclusions

Although several therapies are nowadays available to treat psoriasis, still there is a need for development of new drugs/drug leads with high efficacy and limited side effects. Our findings suggest that LEU may exhibit therapeutic potential in psoriasis via inhibition of the AKT pathway, eventually by regulating epidermal differentiation.

Moreover, the HP cell suspension might be a good source to obtain complex extract that contains primary and secondary metabolites. Such type of extracts possesses several important beneficial properties for skin care, such as antioxidant and anti-inflammatory, hence giving them an opportunity to be incorporated in many cosmeceutical formulations. The HP cell suspension extract besides the content of bioactive LEU, can also be utilized as an active source of ingredients for various topical applications.

## Figures and Tables

**Table 1:** Free energy of binding (_Δ_G, kcal/M) and affinity constant (Ki, µM) for each component to protein structure.

Protein Target	Verbascoside	Leucosceptoside A
AKT	−7.5 kcal/M; 3.2 µM	−7.2 kcal/M; 5.4 µM
PI3K	−9.7 kcal/M; 0.08 µM	−8.8 kcal/M; 0.4 µM
pSTAT1	−8.5 kcal/M; 0.6 µM	−8.2 kcal/M; 1.0 µM
JAK2	−7.5 kcal/M; 3.2 µM	−7.2 kcal/M; 5.4 µM

## Data Availability

Not applicable.

## Supplementary Materials

The following are available online. Table S1: Content of phenylethanoid glycosides in HP cell suspension biomass and crude extract as determined by HPLC, Table S2: Primer sequences used for the RT-qPCR analysis, Figure S1: *Harpagophytum procumbens* (Burch) DC: ex Meisn (HP) cell suspension extract (**A**), pure verbascoside (VER), (**B**) and leucosceptoside A (LEU, (**C**) effect on cell viability in human keratinocytes. HP, VER and LEU at the 24th h of treatment did not affect cell viability in HaCaT cells up to 100 μg/mL and 100 μM respectively, Figures S2: Putative interactions of verbascoside with the binding pocket/domain of JAK2 (PDB: 6BBV), Figure S3: Putative interactions of leucosceptoside A with the binding pocket/domain of JAK2 (PDB: 6BBV), Figure S4: Putative interactions of verbascoside with the binding pocket/domain of pSTAT1 (PDB: 1BF5), Figure S5: Putative interactions of leucosceptoside A with the binding pocket/domain of pSTAT1 (PDB: 1BF5), Figure S6: Putative interactions of verbascoside with the binding pocket/domain of AKT (PDB: 1O6L), Figure S7: Putative interactions of leucosceptoside A with the binding pocket/domain of AKT (PDB: 1O6L), Figure S8: Putative interactions of verbascoside with the binding pocket/domain of PI3K (PDB: 5ITD), Figure S9: Putative interactions of leucosceptoside A with the binding pocket/domain of PI3K (PDB: 5ITD).
