# Supplementary material for: Leucosceptoside A from Devil’s Claw Modulates Psoriasis-like Inflammation via Suppression of the PI3K/AKT Signaling Pathway in Keratinocytes
Source: Molecules. 2021 Nov 20;26(22):7014. doi: 10.3390/molecules26227014 (PMC8618597; doi:10.3390/molecules26227014)
Supplement: Supplementary file 1 [file molecules-26-07014-s001.zip › molecules-1429244-SI.pdf]

Supplementary Material

Leucosceptoside A from Devil's Claw Modulates Psoriasis-like Inflammation *via* Suppression of the PI3K/AKT Signaling Pathway in Keratinocytes

Ivanka K. Koycheva [1,2], Liliya V. Mihaylova [1,2], Monika N. Todorova [1,2], Zhivka P. Balcheva-Sivenova [1,2], Kalina Alipieva [3], Claudio Ferrante [4], Giustino Orlando [4] and Milen I. Georgiev [1,2,*]

[1] Department Plant Cell Biotechnology, Center of Plant Systems Biology and Biotechnology, 4000 Plovdiv, Bulgaria; vkoy4eva@abv.bg (I.K.K.); vasileva.l.vl@gmail.com (L.V.M.); mntodorova@yahoo.com (M.N.T.); sivenova_jivka@abv.bg (Z.P.B.-S.)

[2] Laboratory of Metabolomics, Department of Biotechnology, Institute of Microbiology, Bulgarian Academy of Sciences, 4000 Plovdiv, Bulgaria

[3] Institute of Organic Chemistry with Centre of Phytochemistry, Bulgarian Academy of Sciences, 1113 Sofia, Bulgaria; kalina.alipieva@orgchm.bas.bg

[4] Department of Pharmacy, G.d'Annunzio University, 66100 Chieti, Italy; cferrante@unich.it (C.F.); giustino.orlando@unich.it (G.O.)

* Correspondence: milengeorgiev@gbg.bg

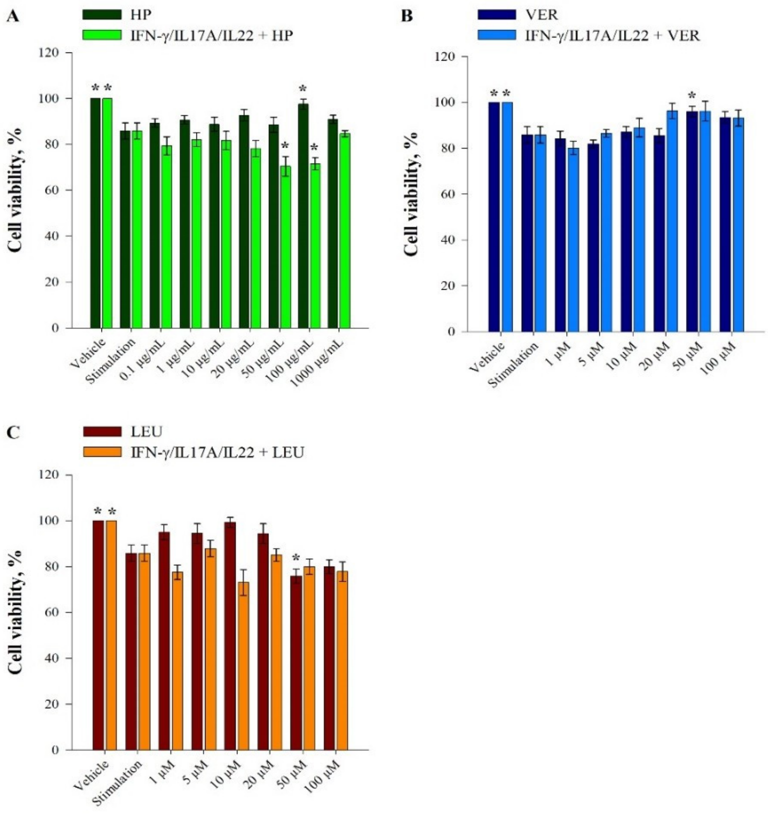

Supplementary Figure S1. *Harpagophytum procumbens* (Burch) DC: ex Meisn (HP) cell suspension extract (**A**), pure verbascoside (VER, **B**) and leucosceptoside A (LEU, **C**) effect on cell viability in human keratinocytes. HP, VER and LEU at the 24th hour of treatment did not affect cell viability in HaCaT cells up to 100 μg/mL and 100 μM respectively.

Cell viability was evaluated through MTT assay. Briefly, HaCaT cells (1 x 10^4 cells/well) were seeded in 96-well plates and cultured for 24 h to reach confluence. Then cells were treated with HP (0.1, 1, 10, 20, 50, 100, 1000 μg/mL), VER (1, 5, 10, 20, 50, 100 μM) or LEU (1, 5, 10, 20, 50, 100 μM) with or without IFN-γ/IL-17A/IL-22 stimulation (1/1/1 ng/mL). On the 24th hour of treatment 10 μL MTT reagent (5 μg/mL), per well, was added and left for 3 h of incubation at 37 °C. Finally, 200 μL of 5% formic acid in isopropanol was used to dissolve the purple formazan crystals formed within the viable cells. Absorbance was measured on a microplate reader Antos Zenyth 340 (Biochrom Ltd, Cambridge, United Kingdom) at 570 nm with reference filter at 620 nm. Cell viability was expressed as a percentage from the non-treated controls, mean ± SEM and *$p < 0.05$ compared to non-treated controls.

Supplementary Table S1. Content of phenylethanoid glycosides in *Harpagophytum procumbens* (HP) cell suspension extract as determined by HPLC

Compound	Retention time (min)	Content of phenylethanoid glycoside in HP biomass (mg/g)	in dry HP extract (mg/g)
Verbascoside	12.132	3.99 ± 0.49	10.94 ± 1.18
Leucosceptoside A	13.257	0.60 ± 0.07	1.64 ± 0.16

Supplementary Table S2. Primer sequences for the RT-qPCR analysis

Target gene (human)	Sequence forward primer (5′ - 3′)	Sequence reverse primer (5′ - 3′)
AKT1	CGAGCTGTTCTTCCACCTGT	TAATGTGCCCGTCCTTGTCC
CCL2	GATCTCAGTGCAGAGGCTCG	TTTGCTTGTCCAGGTGGTCC
CCL20	AGTTGTCTGTGTGCGCAAATCC	TCCAACCCCAGCAAGGTTCT
CHUK	TTCTGTTACCACCTGATGAAAGTCT	ATTGAGAGGCTGGTTTCCGAG
CXCL8	GGTGCAGTTTTGCCAAGGAG	TTCCTTGGGGTCCAGACAGA
DEFB1	TGTCAGCTCAGCCTCCAAAG	TACCACCTGAGGCCATCTCA
DEFB4A	GACTCAGCTCCTGGTGAAGCTC	CTATACCACCAAAAACACCTGGAAG
GAPDH	CCCACTCCTCCACCTTTGAC	TCCTCTTGTGCTCTTGCTGG
IKBKB	TGAGAAGACTGTTGTCCGGC	CACTCTTCTTGGCTGGCTCA
IL6	TGCAATAACCACCCCTGACC	GTGCCCATGCTACATTTGCC
JAK2	CAAAGCAACTGTCATGGCCC	TCTCGCTCGACAGCAAAAGT
MKI67	CGGATCGTCCCAGTGGAAG	TCTCGTGGGCCACATTTTCT
NFKB1	GGCTACACCGAAGCAATTGAA	CAGCGAGTGGGCCTGAGA
NFKBIA	GAAGTGATCCGCCAGGTGAA	CTCACAGGCAAGGTGTAGGG
PI3KCA	GGACCCGATGCGGTTAGAG	ATCAAGTGGATGCCCCACAG
PTGS2	CGACTCCCTTGGGTGTCAAA	TGGCCCTCGCTTATGATCTG
RELA	TTCCAACTGCCCCCAACTTT	TTTGAGTTTCCCCAGCTCCC
S100A7	ACACTCAAGCTGAGAGGTCCA	AAGACATCGGCGAGGTAATTTGT
STAT1	GGATCAGCTGCAGAACTGGT	GAAGGTGCGGTCCCATAACA
STAT3	ACCAACGACCTGCAGCAATA	TCTGCAGCTTCCGTTCTCAG
TUBB	AGCCGTCTTACTCAACTGCC	GTCACCCAGAATGGCAGAA

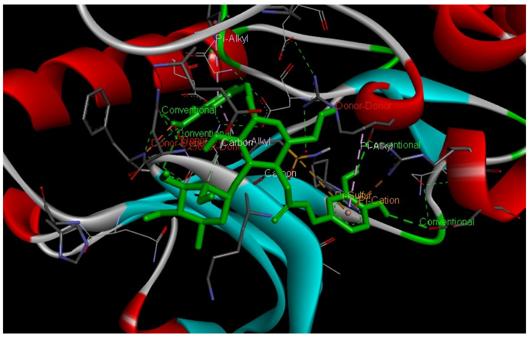

	Name	Visible	Color	Parent	Distance	Category	Types	From	From Chemistry	To	To Chemistry
1	:UNK0:H27 - A:ARG980:O	Yes		Ligand Non-bond Monitor	2,54199	Hydrogen Bond	Conventional Hydrogen Bond	:UNK0:H27	H-Donor	A:ARG980:O	H-Acceptor
2	:UNK0:H26 - A:ASP939:OD2	Yes		Ligand Non-bond Monitor	2,85103	Hydrogen Bond	Conventional Hydrogen Bond	:UNK0:H26	H-Donor	A:ASP939:OD2	H-Acceptor
3	:UNK0:H40 - A:ASN859:OD1	Yes		Ligand Non-bond Monitor	1,74368	Hydrogen Bond	Conventional Hydrogen Bond	:UNK0:H40	H-Donor	A:ASN859:OD1	H-Acceptor
4	A:GLY861:N - :UNK0:O40	Yes		Ligand Non-bond Monitor	3,37685	Hydrogen Bond	Conventional Hydrogen Bond	A:GLY861:N	H-Donor	:UNK0:O40	H-Acceptor
5	:UNK0:C14 - A:LYS857:O	Yes		Ligand Non-bond Monitor	3,31327	Hydrogen Bond	Carbon Hydrogen Bond	:UNK0:C14	H-Donor	A:LYS857:O	H-Acceptor
6	:UNK0:C5 - A:GLU1015:OE1	Yes		Ligand Non-bond Monitor	3,35966	Hydrogen Bond	Carbon Hydrogen Bond	:UNK0:C5	H-Donor	A:GLU1015:OE1	H-Acceptor
7	A:ARG938:NH1 - :UNK0	Yes		Ligand Non-bond Monitor	3,75342	Electrostatic	Pi-Cation	A:ARG938:NH1	Positive	:UNK0	Pi-Orbitals
8	A:D7D1201:S - :UNK0	Yes		Ligand Non-bond Monitor	5,29951	Other	Pi-Sulfur	A:D7D1201:S	Sulfur	:UNK0	Pi-Orbitals
9	:UNK0 - A:ARG980	Yes		Ligand Non-bond Monitor	5,32021	Hydrophobic	Pi-Alkyl	:UNK0	Pi-Orbitals	A:ARG980	Alkyl
10	:UNK0 - A:LEU997	Yes		Ligand Non-bond Monitor	4,86767	Hydrophobic	Pi-Alkyl	:UNK0	Pi-Orbitals	A:LEU997	Alkyl
11	:UNK0 - A:D7D1201:C10	Yes		Ligand Non-bond Monitor	4,4613	Hydrophobic	Pi-Alkyl	:UNK0	Pi-Orbitals	A:D7D1201:C10	Alkyl

Supplementary Figure S2. Putative interactions of verbascoside with the binding pocket/domain of JAK2 (PDB: 6BBV).

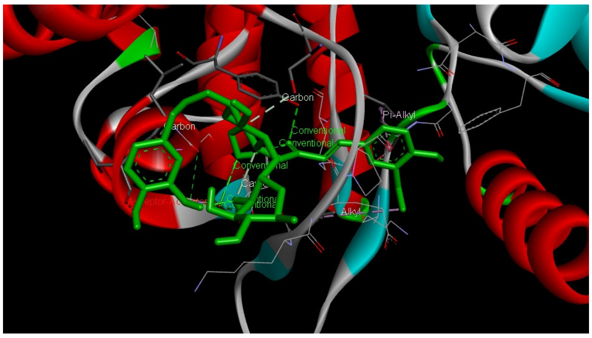

	Name	Visible	Color	Parent	Distance	Category	Types	From	From Chemistry	To	To Chemistry
1	:UNK0:H43 - A:SER1029:OG	Yes		Ligand Non-bond Monitor	2,59124	Hydrogen Bond	Conventional Hydrogen Bond	:UNK0:H43	H-Donor	A:SER1029:OG	H-Acceptor
2	:UNK0:H30 - :UNK0:O17	Yes		Ligand Non-bond Monitor	2,29801	Hydrogen Bond	Conventional Hydrogen Bond	:UNK0:H30	H-Donor	:UNK0:O17	H-Acceptor
3	:UNK0:H41 - :UNK0:O43	Yes		Ligand Non-bond Monitor	1,94185	Hydrogen Bond	Conventional Hydrogen Bond	:UNK0:H41	H-Donor	:UNK0:O43	H-Acceptor
4	A:TYR1021:OH - :UNK0:O17	Yes		Ligand Non-bond Monitor	3,2901	Hydrogen Bond	Conventional Hydrogen Bond	A:TYR1021:OH	H-Donor	:UNK0:O17	H-Acceptor
5	A:SER1029:OG - :UNK0:O30	Yes		Ligand Non-bond Monitor	3,10291	Hydrogen Bond	Conventional Hydrogen Bond	A:SER1029:OG	H-Donor	:UNK0:O30	H-Acceptor
6	:UNK0:C13 - A:SER1016:OG	Yes		Ligand Non-bond Monitor	3,41404	Hydrogen Bond	Carbon Hydrogen Bond	:UNK0:C13	H-Donor	A:SER1016:OG	H-Acceptor
7	:UNK0:C14 - :UNK0:O43	Yes		Ligand Non-bond Monitor	3,30908	Hydrogen Bond	Carbon Hydrogen Bond	:UNK0:C14	H-Donor	:UNK0:O43	H-Acceptor
8	:UNK0:C32 - A:LEU1026:O	Yes		Ligand Non-bond Monitor	3,59838	Hydrogen Bond	Carbon Hydrogen Bond	:UNK0:C32	H-Donor	A:LEU1026:O	H-Acceptor
9	:UNK0:C28 - A:VAL1010	Yes		Ligand Non-bond Monitor	5,03196	Hydrophobic	Alkyl	:UNK0:C28	Alkyl	A:VAL1010	Alkyl
10	:UNK0 - A:LEU997	Yes		Ligand Non-bond Monitor	4,92475	Hydrophobic	Pi-Alkyl	:UNK0	Pi-Orbitals	A:LEU997	Alkyl

Supplementary Figure S3. Putative interactions of leucosceptoside A with the binding pocket/domain of JAK2 (PDB: 6BBV).

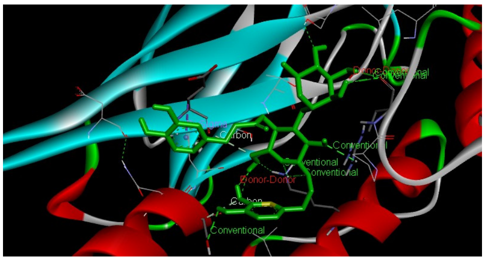

	Name	Visible	Color	Parent	Distance	Category	Types	From	From Chemistry	To	To Chemistry
1	:UNK0:H4...	Yes		Ligand Non-bond Monitor	2,41369	Hydrogen Bond	Conventional Hydrogen Bond	:UNK0:H42	H-Donor	A:ARG482:O	H-Acceptor
2	:UNK0:H4...	Yes		Ligand Non-bond Monitor	2,09306	Hydrogen Bond	Conventional Hydrogen Bond	:UNK0:H43	H-Donor	A:ARG482:O	H-Acceptor
3	:UNK0:H3...	Yes		Ligand Non-bond Monitor	2,59669	Hydrogen Bond	Conventional Hydrogen Bond	:UNK0:H39	H-Donor	A:SER315:OG	H-Acceptor
4	A:LYS240...	Yes		Ligand Non-bond Monitor	2,24952	Hydrogen Bond	Conventional Hydrogen Bond	A:LYS240:HZ3	H-Donor	:UNK0:O12	H-Acceptor
5	A:LYS240...	Yes		Ligand Non-bond Monitor	2,29319	Hydrogen Bond	Conventional Hydrogen Bond	A:LYS240:HZ3	H-Donor	:UNK0:O30	H-Acceptor
6	A:ARG48...	Yes		Ligand Non-bond Monitor	2,64303	Hydrogen Bond	Conventional Hydrogen Bond	A:ARG482:HH12	H-Donor	:UNK0:O41	H-Acceptor
7	:UNK0:C2...	Yes		Ligand Non-bond Monitor	3,71717	Hydrogen Bond	Carbon Hydrogen Bond	:UNK0:C28	H-Donor	A:GLU449:O	H-Acceptor
8	A:SER31...	Yes		Ligand Non-bond Monitor	3,24937	Hydrogen Bond	Carbon Hydrogen Bond	A:SER315:CA	H-Donor	:UNK0:O40	H-Acceptor
9	A:GLU44...	Yes		Ligand Non-bond Monitor	3,76052	Hydrophobic	Pi-Sigma	A:GLU449:CB	C-H	:UNK0	Pi-Orbitals

Supplementary Figure S4. Putative interactions of verbascoside with the binding pocket/domain of pSTAT1 (PDB: 1BF5).

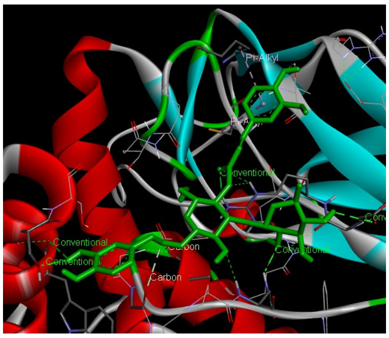

	Name	ID	Visible	Color	Parent	X	Y	Z	Distance	Donor Atom	Acceptor Atom
1	:UNK0:H41 - A:SER507:OG	0	Yes		<HBondMonitor>	86,8865	47,4...	79,6...	2,03972	H41	OG
2	A:TRP239:HE1 - A:ALA254:O	0	Yes		<HBondMonitor>	71,3275	44,0...	71,0...	2,03559	HE1	O
3	A:GLN243:HE22 - :UNK0:O43	0	Yes		<HBondMonitor>	70,5925	48,0...	76,3...	2,49724	HE22	O43
4	A:GLN243:HE22 - A:GLU320:O	0	Yes		<HBondMonitor>	69,038	48,5...	76,2...	2,49295	HE22	O
5	A:GLY249:HN - A:ALA246:O	0	Yes		<HBondMonitor>	78,64	47,7...	80,5...	1,88777	HN	O
6	A:ASN253:HN - :UNK0:O42	0	Yes		<HBondMonitor>	76,302	47,1...	74,4...	2,14707	HN	O42
7	A:ASN253:HD22 - :UNK0:O43	0	Yes		<HBondMonitor>	72,1145	47,2...	75,7...	2,21695	HD22	O43
8	A:ARG321:HE - :UNK0:O15	0	Yes		<HBondMonitor>	73,9285	52,5...	77,0...	2,49097	HE	O15
9	A:ARG321:HE - :UNK0:O17	0	Yes		<HBondMonitor>	73,7	53,1...	78,0...	2,37405	HE	O17
10	A:ARG321:HH22 - :UNK0:O17	0	Yes		<HBondMonitor>	74,4415	52,6...	78,7...	2,01016	HH22	O17
11	A:THR342:HG1 - :UNK0:O26	0	Yes		<HBondMonitor>	69,7625	61,0...	76,7...	2,45325	HG1	O26
12	A:ARG346:HH12 - A:GLU320:OE2	0	Yes		<HBondMonitor>	66,576	49,4...	69,6...	2,2219	HH12	OE2
13	A:ARG405:HE - A:GLU403:OE2	0	Yes		<HBondMonitor>	67,2285	60,7...	73,6...	1,78833	HE	OE2
14	A:ARG405:HH22 - A:GLU403:OE2	0	Yes		<HBondMonitor>	67,2705	60,2...	72,6...	2,03134	HH22	OE2
15	A:SER507:HN - A:SER503:O	0	Yes		<HBondMonitor>	88,646	47,9...	82,4...	1,83293	HN	O
16	A:SER507:HN - A:TRP504:O	0	Yes		<HBondMonitor>	87,4265	48,6...	82,0...	2,28878	HN	O

Supplementary Figure S5. Putative interactions of leucosceptoside A with the binding pocket/domain of pSTAT1 (PDB: 1BF5).

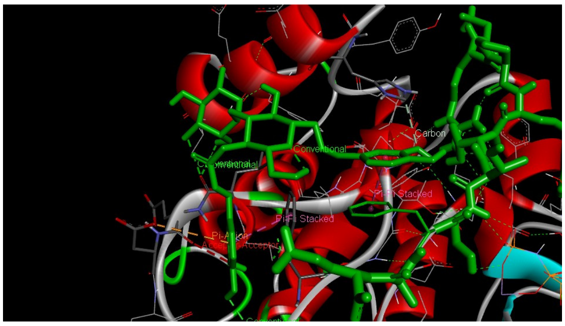

	Name	Visible	Color	Parent	Distance	Category	Types	From	From Chemistry	To	To Chemistry
1	:UNK0 - A...	Yes		Ligand Non-bond Monitor	4,11943	Hydrophobic	Pi-Pi Stac...	:UNK0	Pi-Orbitals	A:PHE310	Pi-Orbitals
2	:UNK0 - C...	Yes		Ligand Non-bond Monitor	4,04866	Hydrophobic	Pi-Pi Stac...	:UNK0	Pi-Orbitals	C:PHE10	Pi-Orbitals
3	:UNK0:H2...	Yes		Ligand Non-bond Monitor	2,64782	Hydrogen Bond	Conventi...	:UNK0:H26	H-Donor	A:LYS308:O	H-Acceptor
4	:UNK0:H2...	Yes		Ligand Non-bond Monitor	2,01912	Hydrogen Bond	Conventi...	:UNK0:H29	H-Donor	:UNK0:O12	H-Acceptor
5	:UNK0:H4...	Yes		Ligand Non-bond Monitor	2,4771	Hydrogen Bond	Conventi...	:UNK0:H42	H-Donor	A:LEU322:O	H-Acceptor
6	:UNK0:H4...	Yes		Ligand Non-bond Monitor	2,75792	Hydrogen Bond	Conventi...	:UNK0:H43	H-Donor	A:LEU322:O	H-Acceptor
7	A:ASP32...	Yes		Ligand Non-bond Monitor	4,72041	Electrostatic	Pi-Anion	A:ASP32...	Negative	:UNK0	Pi-Orbitals
8	A:HIS355...	Yes		Ligand Non-bond Monitor	3,33762	Hydrogen Bond	Carbon H...	A:HIS35...	H-Donor	:UNK0:O40	H-Acceptor

Supplementary Figure S6. Putative interactions of verbascoside with the binding pocket/domain of AKT (PDB: 1O6L).

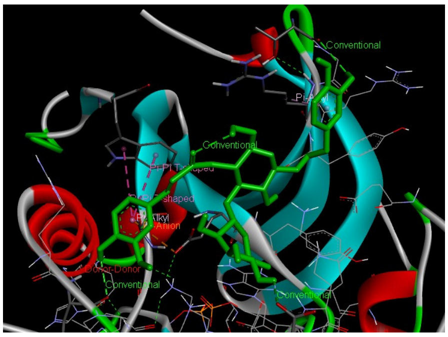

	Name	Visible	Color	Parent	Distance	Category	Types	From	From Chemistry	To	To Chemistry
1	:UNK0:H2...	Yes		Ligand Non-bond Monitor	2,56422	Hydrogen Bo...	Conventi...	:UNK0:H26	H-Donor	A:HIS209:O	H-Acceptor
2	:UNK0:H4...	Yes		Ligand Non-bond Monitor	2,71149	Hydrogen Bo...	Conventi...	:UNK0:H45	H-Donor	A:ASP286:OD2	H-Acceptor
3	:UNK0:H3...	Yes		Ligand Non-bond Monitor	2,67967	Hydrogen Bo...	Conventi...	:UNK0:H30	H-Donor	:UNK0:O17	H-Acceptor
4	:UNK0:H4...	Yes		Ligand Non-bond Monitor	3,04768	Hydrogen Bo...	Conventi...	:UNK0:H41	H-Donor	A:THR174:O	H-Acceptor
5	A:GLU23...	Yes		Ligand Non-bond Monitor	4,50848	Electrostatic	Pi-Anion	A:GLU230:OE2	Negative	:UNK0	Pi-Orbitals
6	:UNK0 - A...	Yes		Ligand Non-bond Monitor	5,06586	Hydrophobic	Pi-Pi T-sh...	:UNK0	Pi-Orbitals	A:TRP479	Pi-Orbitals
7	A:TRP47...	Yes		Ligand Non-bond Monitor	5,09708	Hydrophobic	Pi-Pi T-sh...	A:TRP479	Pi-Orbitals	:UNK0	Pi-Orbitals
8	:UNK0 - A...	Yes		Ligand Non-bond Monitor	3,83691	Hydrophobic	Pi-Alkyl	:UNK0	Pi-Orbitals	A:ALA214	Alkyl
9	:UNK0 - A...	Yes		Ligand Non-bond Monitor	3,87357	Hydrophobic	Pi-Alkyl	:UNK0	Pi-Orbitals	A:ARG176	Alkyl

Supplementary Figure S7. Putative interactions of leucosceptoside A with the binding pocket/domain of AKT (PDB: 1O6L).

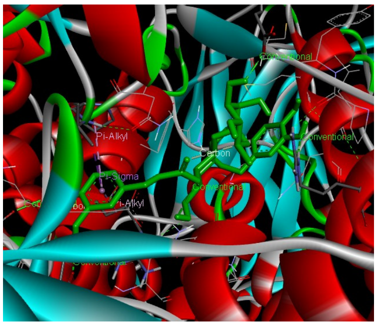

	Name	Visible	Color	Parent	Distance	Category	Types	From	From Chemistry	To	To Chemistry
1	:UNK0 - A...	Yes		Ligand Non-bond Monitor	4,11943	Hydrophobic	Pi-Pi Stac...	:UNK0	Pi-Orbitals	A:PHE310	Pi-Orbitals
2	:UNK0 - C...	Yes		Ligand Non-bond Monitor	4,04866	Hydrophobic	Pi-Pi Stac...	:UNK0	Pi-Orbitals	C:PHE10	Pi-Orbitals
3	:UNK0:H2...	Yes		Ligand Non-bond Monitor	2,64782	Hydrogen Bond	Conventi...	:UNK0:H26	H-Donor	A:LYS308:O	H-Acceptor
4	:UNK0:H2...	Yes		Ligand Non-bond Monitor	2,01912	Hydrogen Bond	Conventi...	:UNK0:H29	H-Donor	:UNK0:O12	H-Acceptor
5	:UNK0:H4...	Yes		Ligand Non-bond Monitor	2,4771	Hydrogen Bond	Conventi...	:UNK0:H42	H-Donor	A:LEU322:O	H-Acceptor
6	:UNK0:H4...	Yes		Ligand Non-bond Monitor	2,75792	Hydrogen Bond	Conventi...	:UNK0:H43	H-Donor	A:LEU322:O	H-Acceptor
7	A:ASP32...	Yes		Ligand Non-bond Monitor	4,72041	Electrostatic	Pi-Anion	A:ASP32...	Negative	:UNK0	Pi-Orbitals
8	A:HIS355...	Yes		Ligand Non-bond Monitor	3,33762	Hydrogen Bond	Carbon H...	A:HIS35...	H-Donor	:UNK0:O40	H-Acceptor

Supplementary Figure S8. Putative interactions of verbascoside with the binding pocket/domain of PI3K (PDB: 5ITD).

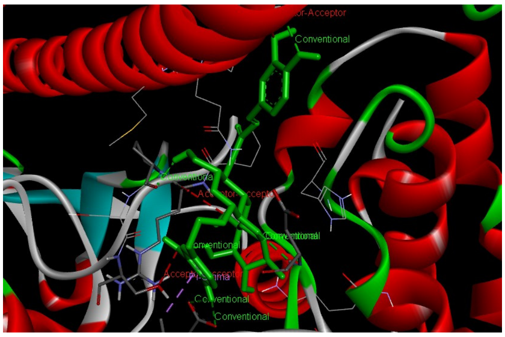

	Name	Visible	Color	Parent	Distance	Category	Types	From	From Chemistry	To	To Chemistry
1	:UNK0:H4...	Yes		Ligand Non-bond Monitor	2,44422	Hydrogen Bo...	Conventional Hydrogen Bond	:UNK0:H44	H-Donor	:UNK0:O41	H-Acceptor
2	:UNK0:H3...	Yes		Ligand Non-bond Monitor	2,05115	Hydrogen Bo...	Conventional Hydrogen Bond	:UNK0:H30	H-Donor	A:PRO5:O	H-Acceptor
3	:UNK0:H4...	Yes		Ligand Non-bond Monitor	2,07774	Hydrogen Bo...	Conventional Hydrogen Bond	:UNK0:H42	H-Donor	:UNK0:O7	H-Acceptor
4	:UNK0:H4...	Yes		Ligand Non-bond Monitor	3,05817	Hydrogen Bo...	Conventional Hydrogen Bond	:UNK0:H40	H-Donor	A:GLU710:OE1	H-Acceptor
5	:UNK0:H4...	Yes		Ligand Non-bond Monitor	2,99354	Hydrogen Bo...	Conventional Hydrogen Bond	:UNK0:H40	H-Donor	A:GLU710:OE2	H-Acceptor
6	A:ASP84...	Yes		Ligand Non-bond Monitor	2,76805	Hydrogen Bo...	Conventional Hydrogen Bond	A:ASP843:HN	H-Donor	:UNK0:O42	H-Acceptor
7	B:SER474...	Yes		Ligand Non-bond Monitor	2,72245	Hydrogen Bo...	Conventional Hydrogen Bond	B:SER474:HG	H-Donor	:UNK0:O26	H-Acceptor
8	A:ILE713...	Yes		Ligand Non-bond Monitor	3,70008	Hydrophobic	Pi-Sigma	A:ILE713:CG2	C-H	:UNK0	Pi-Orbitals

Supplementary Figure S9. Putative interactions of leucosceptoside A with the binding pocket/domain of PI3K (PDB: 5ITD).
